# Disruption of pyruvate phosphate dikinase in *Brucella ovis* PA CO_2_-dependent and independent strains generates attenuation in the mouse model

**DOI:** 10.1186/s13567-020-00824-7

**Published:** 2020-08-14

**Authors:** Nieves Vizcaíno, Lara Pérez-Etayo, Raquel Conde-Álvarez, Maite Iriarte, Ignacio Moriyón, Amaia Zúñiga-Ripa

**Affiliations:** 1 Departamento de Microbiología Y Genética, Edificio Departamental, Universidad de Salamanca, Edificio Departamental, Plaza Doctores de la Reina s/n, Salamanca, 37007 Spain; 2 Instituto de Investigación Biomédica de Salamanca (IBSAL), Hospital Universitario de Salamanca, Paseo de San Vicente 52-182, 37007 Salamanca, Spain; 3 Instituto de Salud Tropical (ISTUN), Instituto de Investigación Sanitaria de Navarra (IdiSNA), Departamento de Microbiología y Parasitología, Universidad de Navarra, Calle Irunlarrea 1, 31008 Pamplona, Spain

**Keywords:** *Brucella ovis*, metabolism, gluconeogenesis, pyruvate phosphate dikinase, attenuation, laboratory models, vaccine, virulence

## Abstract

*Brucella ovis* is a non-zoonotic rough *Brucella* that causes genital lesions, abortions and increased perinatal mortality in sheep and is responsible for important economic losses worldwide. Research on virulence factors of *B. ovis* is necessary for deciphering the mechanisms that enable this facultative intracellular pathogen to establish persistent infections and for developing a species-specific vaccine, a need in areas where the cross-protecting ovine smooth *B. melitensis* Rev1 vaccine is banned. Although several *B. ovis* virulence factors have been identified, there is little information on its metabolic abilities and their role in virulence. Here, we report that deletion of pyruvate phosphate dikinase (PpdK, catalyzing the bidirectional conversion pyruvate ⇌ phosphoenolpyruvate) in *B. ovis* PA (virulent and CO_2_-dependent) impaired growth in vitro. In cell infection experiments, although showing an initial survival higher than that of the parental strain, this *ppdK* mutant was unable to multiply. Moreover, when inoculated at high doses in mice, it displayed an initial spleen colonization higher than that of the parental strain followed by a marked comparative decrease, an unusual pattern of attenuation in mice. A homologous mutant was also obtained in a *B. ovis* PA CO_2_-independent construct previously proposed for developing *B. ovis* vaccines to solve the problem that CO_2_-dependence represents for large scale production. This CO_2_-independent *ppdK* mutant reproduced the growth defect in vitro and the multiplication/clearance pattern in mouse spleens, and is thus an interesting vaccine candidate for the immunoprophylaxis of *B. ovis* ovine brucellosis.

## Introduction

Brucellosis is an infectious disease caused by several species of the intracellular gram-negative pathogen *Brucella* spp. This disease affects domestic and wild animals and can be transmitted to humans, producing important economic losses and human suffering in many countries throughout the world [1]. Currently, these bacteria are grouped in a single genus with up to 12 nominal species that often show host preference (https://lpsn.dsmz.de/genus/brucella). The zoonotic brucellae that infect cattle (*B. abortus*), swine (*B. suis* biovars 1, 2, and 3) and goats and sheep (*B. melitensis*) carry a smooth lipopolysaccharide (LPS) and have deserved greater attention because of their great impact on public health and animal production. Sheep can also be infected by *B. ovis*, a non-zoonotic species that causes ovine epididymitis and is naturally rough (i.e. bears an LPS lacking the O-polysaccharide section) [2, 3]. *B. ovis* is considered one of the most important causes of ovine infertility and has a significant economic impact on sheep husbandry [4, 5].

Animal vaccination is the most suitable method for controlling brucellosis in areas with moderate to high prevalence of the disease. Since sheep brucellosis can be caused by either *B. melitensis* or *B. ovis* and no specific vaccine against *B. ovis* is available, the attenuated vaccine *B. melitensis* Rev1 has been used to control infections by both bacteria. However, this vaccine has several drawbacks [6–8], among them its virulence for humans and an induction of persistent antibodies against the LPS O-polysaccharide [9]. Since this is the antigen used in the diagnosis of *B. melitensis* infections, those antibodies hamper the discrimination of Rev1 vaccinated and *B. melitensis* infected animals. Owing to this drawback, Rev1 is banned in regions or countries where *B. melitensis* has been eradicated [10], thus favoring the emergence of *B. ovis* infections. Therefore, research on vaccines on a *B. ovis* background is of great interest as such species-specific vaccines would neither interfere in *B. melitensis* serological tests nor cause human infections [11–13].

Current live attenuated brucellosis vaccines reproduce closely the cell invasion, intracellular trafficking and antigen presentation of virulent brucellae [14] and are thus the best vaccines available against *B. abortus* and *B. melitensis* [15]. Indeed, the development of new attenuated vaccines depends on an understanding of the virulence factors involved in infection, a topic that is delayed in *B. ovis* with respect to its smooth zoonotic counterparts. Several *B. ovis* attenuated mutants in classic *Brucella* virulence factors, outer membrane proteins, core LPS glycosyltransferases and an ABC transporter have been described [16–19], some of them providing interesting results as potential vaccines [18, 20–23]. However, recent works emphasize the relevance of bacterial metabolism in the virulence of smooth *Brucella* species [24, 25], an aspect of the biology of the parasite yet to be explored in *B. ovis*.

In *B. abortus* 2308 W, we have shown that disruption of pyruvate phosphate dikinase (PpdK) (catalyzing the bidirectional conversion ATP + pyruvate + Pi ⇌ AMP + phosphoenolpyruvate + PPi) severely affects growth on gluconeogenic substrates and causes attenuation in mice [26], strongly suggesting an important role for the phosphoenolpyruvate (PEP)-Tricarboxylic Acid Cycle (TCA) connections in virulence. Considering the great homogeneity of the *Brucella* genus, it seemed plausible that this metabolic step is also important in the virulence of other brucellae and, in keeping with this hypothesis, we found that attenuation occurs in a double PckA-PpdK mutant of *B. suis* 513, a more prototrophic biovar 5 strain that uses both PpdK and phosphoenolpyruvate carboxykinase (PckA; ATP + oxaloacetate → ADP + CO_2_ + PEP) for PEP synthesis [27]. Accordingly, the aim of this research was to investigate whether genes coding for PpdK and PckA are present in *B. ovis* PA and to determine their involvement in metabolism and virulence. Additionally, since the CO_2_ requirement of *B. ovis* represents a significant obstacle for large-scale production of a live attenuated vaccine, we explored the possibilities of combining deletion of those metabolic genes with genetic modifications of the *B. ovis* carbonic anhydrases required for CO_2_-independent growth that we have recently deciphered [28].

## Materials and methods

### Bacterial strains and culture conditions

*B. ovis* PA is a virulent strain that, like most *B. ovis* strains, requires CO_2_ for growth. It was used as the parental strain to obtain the recombinant *B. ovis* strains used in this work (Table 1). *B. ovis* strains were cultivated at 37 °C under a 5% CO_2_ atmosphere, except for the selection and characterization of CO_2_-independent strains. Tryptic soy agar (TSA) or tryptic soy broth (TSB) (Pronadisa-Laboratorios Conda), both supplemented with 0.3% yeast extract (YE) (Pronadisa-Laboratorios Conda) and 5% horse serum (HS) (Gibco-Life Technologies), were used as solid (TSA-YE-HS) and liquid (TSB-YE-HS) culture medium, respectively.

**Table 1:** Most relevant bacterial strains used in this work.

*Brucella ovis* strains^a^	Main characteristics	Source
*B. ovis* PA	Virulent parental strain; CO_2_-dependent	BCCN (76-250)
*Bov*::CA	*B. ovis* PA carrying Tn7_Ba2308W_CAI_Ba2308W_CAII; CO_2_-independent	This work
*Bov*Δ*ppdK*	Derived from *B. ovis* PA; *ppdK* deletion; CO_2_-dependent	This work
*Bov*Δ*ppdK* pAZI-19	*Bov*Δ*ppdK* complemented in trans with *ppdK* gene cloned into pRH001; CO_2_-dependent	This work
*Bov*::CAΔ*ppdK*	Derived from *Bov*::CA; *ppdK* deletion; CO_2_-independent	This work
*Bov*::CAΔ*ppdK* pAZI-19	*Bov*::CAΔ*ppdK* complemented in trans with *ppdK* gene cloned into pRH001; CO_2_-independent	This work

^a^The *B. ovis* recombinant strains derive from *B. ovis* PA, which was obtained from BCCN (*Brucella* Culture Collection Nouzilly, Institut National de la Recherche Agronomique, Nouzilly, France).

To study the metabolic phenotype of the *B. ovis* strains, the gluconeogenic minimal medium of Gerhardt and Wilson (referred to here as Gerhardt) was used [29]. Growth was also tested on this medium supplemented with 5% horse serum (Gerhardt-HS), 5% horse serum and methionine 1 mM (Gerhardt-HS-meth) or the latter adding a mixture of vitamins provided by the RPMI supplement R7256 (Sigma Aldrich) (Gerhardt-HS-meth-vit). The composition of the different defined media is detailed in Additional file 1. The pH was adjusted to 7.

When required, media were supplemented with 5% sucrose (Sigma) or with kanamycin (Km; 50 μg/mL) and/or nalidixic acid (Nal; 25 μg/mL) (Sigma). All strains were stored at −80 °C in TSB-YE-HS with 7% DMSO.

### DNA manipulations and analysis

Genomic sequences were obtained from the database National Center for Biotechnology Information (NCBI) and Kyoto Encyclopedia of Genes and Genomes (KEGG). Searches for DNA and protein homologies were carried out using NCBI BLAST [30]. Sequence alignments were performed with Clustal Omega [31, 32]. Plasmid DNA was extracted with QIAprep Spin Miniprep (Qiagen). PCR amplification for verification of genetically modified *B. ovis* strains was performed with Red Taq DNA polymerase master mix (VWR, Leuven, Belgium).

### Plasmids and mutagenesis

Construction of the *ppdK* mutants was done using previously described plasmid [26] and strategy [16, 18]. Briefly, to obtain the in-frame *ppdK* mutant (*Bov*Δ*ppdK)*, the suicide plasmid pMZI-2 was introduced into *B. ovis* PA and both the deletion mutant and the sibling revertant strain generated by allelic exchange were selected by sucrose resistance and Km sensitivity and by PCR using external and internal primers to the deleted fragment. The mutation generated resulted in the loss of the 93% of *ppdK* (BOV_RS02525 locus of the *B. ovis* 63/290 genome; accession numbers NC_009505 and NC_009504 for chromosome I and II, respectively).

The CO_2_-independent strain *Bov*::CA was obtained using previously described strategy [28]. Briefly, the genes coding for the CAI and CAII carbonic anhydrases of the CO_2_-independent strain *B. abortus* 2308 W were cloned into vector pUC18R6KT-miniTn7-Km^R^ [33]. The recombinant plasmid was introduced in *B. ovis* PA and the desired *Bov*::CA strain (containing both CA genes inserted in the genome between *glmS* and *recG*) was selected according to previously described procedures [28]. Kanamycin resistance cassette was deleted following the protocol set up by Martínez-Gómez et al. [34]. Finally, to obtain the CO_2_-independent *ppdK* mutant (*Bov*::CAΔ*ppdK*), the suicide plasmid pMZI-2 was introduced into *Bov*::CA following the procedure described above.

*In trans* complementation of both mutants with wild-type *ppdK* (*Bov*Δ*ppdK* pAZI-19 and *Bov*::CAΔ*ppdK* pAZI-19) was performed with plasmid pAZI-19 [26].

### Bacterial growth evaluation

Growth curves in TSB-YE-HS were stablished for all strains from initial bacterial suspensions with an optical density (OD_600_) of 0.05 in 30 mL of culture medium that were incubated under agitation (120 rpm). OD_600_ values were determined through a 120-h period.

Growth evaluation in minimal media was performed as follows. CO_2_-independent *B. ovis* PA was inoculated into 10 mL of TSB-YE-HS in a 50-mL flask and incubated at 37 °C with orbital shaking for 18 h. These exponentially growing bacteria were harvested by centrifugation, resuspended in 5 mL of each test medium at an OD_600_ of 0.1, and incubated at 37 °C with orbital shaking for 18 h. Then, these preconditioned bacteria were harvested by centrifugation, resuspended at an OD_600_ of 0.1 in the same test medium in Bioscreen multiwell plates (200 μL/well), and incubated in a Bioscreen C (Lab Systems) apparatus with continuous shaking at 37 °C. Absorbance values at 420–580 nm were automatically recorded at 0.5-h intervals over a 300-h period. All experiments were performed in triplicate. Controls with medium and no bacteria were included in all experiments.

### Cell infection assays

Internalization and intracellular survival in J774.A1 murine macrophage-like cell line (DSMZ ACC170) and epithelial HeLa cells (ATCC CCL-2TM) were evaluated according to previously described protocols [35]. Briefly, cell lines were cultured for 24 h in 96-well sterile plates at 37 °C under a 5% CO_2_ atmosphere (2 × 10^4^ J744.A1 cells/well or 1.5 x 10^4^ HeLa cells/well) and infected with the different *B. ovis* strains (4 x 10^6^ CFU/well for macrophages or 8 x 10^6^ CFU/well for HeLa cells). Bacteria were discarded after 2-h incubation, and gentamycin was added to kill extracellular bacteria. After 1-h incubation, three wells per *B. ovis* strain were washed to remove gentamycin, eukaryotic cells were lysed by incubation with H_2_O, and intracellular bacteria were enumerated by platting serial dilutions of the content of the wells on TSA-YE-HS [t0 post-infection (p.i.)]. The remaining wells were maintained in the presence of gentamycin and three wells per strain were similarly processed to determine intracellular bacteria at t20 and t44 p.i. The results were expressed as mean ± SD of the log CFU/well at each time point and are representative of three independent experiments.

### Mouse assays and ethics statement

In vivo virulence experiments were performed with female 6-week-old BALB/c mice (Charles River Laboratories, France), received 1 week before, that were kept with water and food ad libitum at registered facilities of the University of Salamanca (PAE SA-001). All procedures involving animals were designed according to Spanish and European laws regarding the use of animals in research (RD 53/2013 and directive 2010/63/UE). They were approved by the Bioethics Committee of the University of Salamanca and authorized by the competent authority of “Junta de Castilla y León”, Spain. Microbiological protocols were approved by the Biosecurity Committees of the Universities of Salamanca and Navarra.

In the first experiment, mice were inoculated by the intraperitoneal route with 10^6^ or 10^8^ CFU of *B. ovis* PA or *Bov*Δ*ppdK* in 200 µL of phosphate buffered saline (PBS). Bacterial splenic colonization was determined in five mice per group at weeks 3 and 11 p.i. as previously described [20]. These time points were chosen because, in the parental strain week 3 corresponds to the acute phase of infection (highest numbers of splenic bacteria) while week 11 corresponds to a point of the chronic phase when a decrease of CFU in spleen is observed [17, 21].

Subsequently, the level of splenic infection was determined at weeks 1, 3, 5, 7 and 11 in mice inoculated with 10^8^ CFU of the CO_2_-dependent (*B. ovis* PA and *Bov*Δ*ppdK*) and CO_2_-independent (*Bov*::CA and *Bov*::CAΔ*ppdK*) strains. The complemented mutant strains (*Bov*Δ*ppdK* pAZI-19 and *Bov*::CAΔ*ppdK* pAZI-19) were also evaluated at weeks 1, 3, 7 and 11.

### Statistical analysis

Statistical comparisons between means were performed with one-way ANOVA and the Fisher´s Least Significant Differences test of GraphPad Prism Software (GraphPad Software Inc., San Diego, USA). Statistically significant differences (*P *< 0.01) were established with a 99% confidence interval.

## Results

### Comparison of *pckA* and *ppdK* orthologues

Concerning *pckA*, *B. ovis* 63/290 BOV_RS09880 is annotated as a pseudogene that hypothetically encodes a protein of 458 amino acids, shorter than the 536-amino acid PckA proteins encoded by *Agrobacterium tumefaciens* C58 (ATU_RS00170) and *B. suis* 513 (genome without annotation, contig accession number ACBK01000073), both previously shown to be functional [27, 36]. The *B. ovis* 63/290 putative PckA enzyme keeps the specific domain (IGGTSYAGE-KKS; 190 to 202) required for its activity (Additional file 2). The phosphate-binding (G–G-GKT) and adenine-binding (IIML–D) consensus sites of ATP-dependent proteins (residues 233 to 243 and 345 to 351, respectively) as well as a metal ion binding (G—EG) site (residues 265 to 271) [36, 37] are also conserved (Additional file 2). However, these domains are also present in the protein encoded by the *pckA* ortholog of *B. abortus* 2308 W (BAB1_2091), which encodes an inactive protein of 491 amino acids because of a premature stop codon [26] (Additional file 2). Similar to *B. abortus*, deletion of a cytosine at position 1523 in *B. ovis pckA* alters the reading frame and thus only the first 418 amino acids are conserved when compared to *B. suis* 513 (Additional file 2). Accordingly, PckA is not expected to be functional in *B. ovis*, as previously suggested by Tsolis et al. [38].

A search of the *B. ovis* 63/290 genome revealed that the locus BOV_RS02525 putatively encodes PpdK. The predicted protein has 887 amino acids, where residues 19 to 376 contain a PEP-binding domain and amino acids 530 to 883 form the TIM-barrel domain characteristic of PEP-utilizing enzymes. When comparing this protein with its homologues in *B. abortus* 2308 W and *B. suis* 513, both functional [26, 27], only 3 amino acid changes were found: A177V, V312E, C857Y (Additional file 2). Also, an alanine present at position 605 in *B. suis* 513 and *B. ovis* 63/290 is substituted by a glycine in *B. abortus* 2308 W (Additional file 2). Therefore, PpdK should also be functional in *B. ovis* and, accordingly, *ppdK* mutants were constructed in CO_2_-dependent and CO_2_-independent virulent *B. ovis* PA.

### Growth characteristics of *B. ovis* mutants

**Figure 1:**
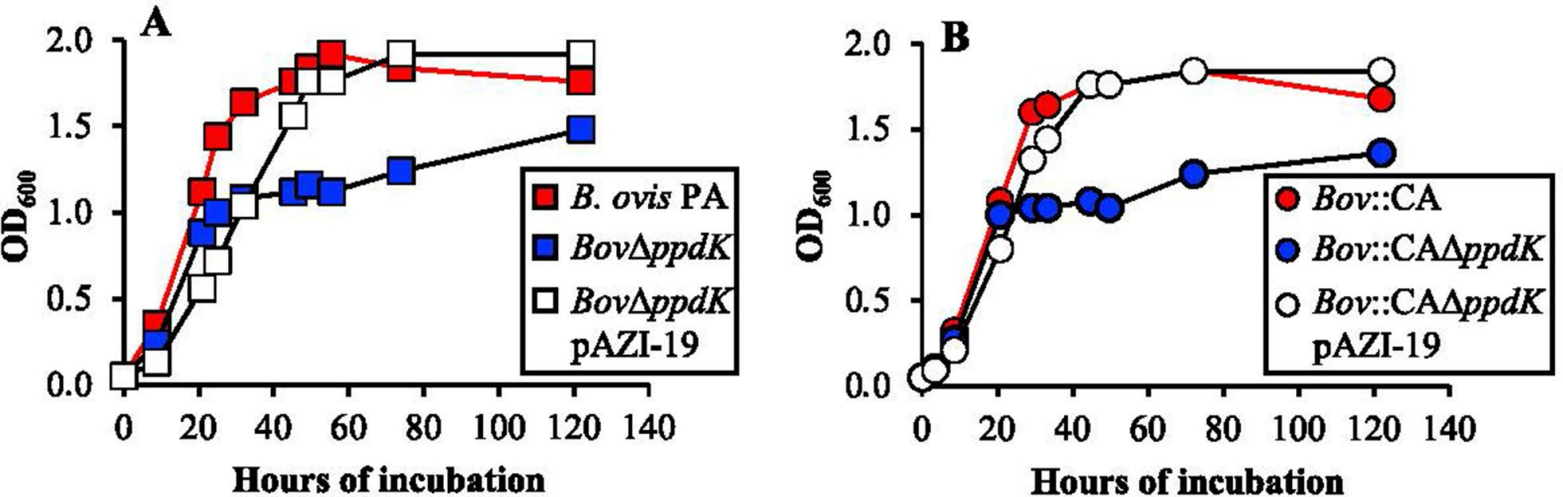
**Growth of the** ***B. ovis*** **strains in TSB-YE-HS**. Growth was evaluated by measuring the evolution over time of the OD_600_ values in TSB-YE-HS liquid medium of CO_2_-dependent strains (**A**) and CO_2_-independent strains (**B**). Results are representative of two independent experiments.

Growth in TSB-YE-HS showed no differences between the CO_2_-dependent and -independent strains (Figure  1). However, although both *ppdK* mutants did not show a marked delayed growth during the first 20 h of incubation, their growth drastically slowed down after that moment, with comparatively reduced maximum OD_600_ scores when compared to their parental strains (Figure  1). Since complementation with plasmid pAZI-19 carrying *ppdK* restored the wild-type phenotype (Figure  1), these results clearly indicate a role for PpdK in *B. ovis* PA metabolism under these in vitro conditions.

As indicated in the Introduction, PpdK catalyzes a bidirectional reaction that can thus be involved in gluconeogenesis when bacteria grow on 3 and 4 C substrates, a function that should be shared by *B. ovis* PpdK [26, 27]. However, although *B. abortus*, *B. melitensis* and *B. suis* grow on glutamate-lactate-glycerol (Gerhardt’s medium) [26, 27, 39], to investigate the gluconeogenic role of PpdK in *B. ovis* PA it was first necessary to examine the ability of this strain to grow in Gerhardt’s medium (Additional file 1). Among brucellae *B. ovis* is notoriously fastidious and requires serum even in rich media. Thus, a first attempt was made by supplementing Gerhardt’s medium with 5% horse serum (Gerhardt-HS, Additional file 1). However, *B. ovis* PA did not grow in this medium. Some *Brucella* strains require methionine to grow in minimal media [25], but when this amino acid was also added to Gerhardt-HS (Gerhardt-HS-meth, Additional file 1) the combination did not support growth of *B. ovis* PA either. Finally, since brucellae show variable auxotrophy for some vitamins, the effect of the vitamin-rich RMPI supplement R7256 was also tested (Gerhardt-HS-meth-vit, Additional file 1). Again, no growth was observed in this medium. These results, that show that *B. ovis* PA is more auxotrophic than other brucellae and are in agreement with the comparative genome degradation of this species [38], preclude to assess whether the growth defect observed in rich medium corresponds to a shutdown of fueling of C precursors to the TCA (i.e. a catabolic role) or, on the contrary, to a gluconeogenic flux from TCA to the triose-phosphate pathway.

### Virulence in cellular models

**Figure 2:**
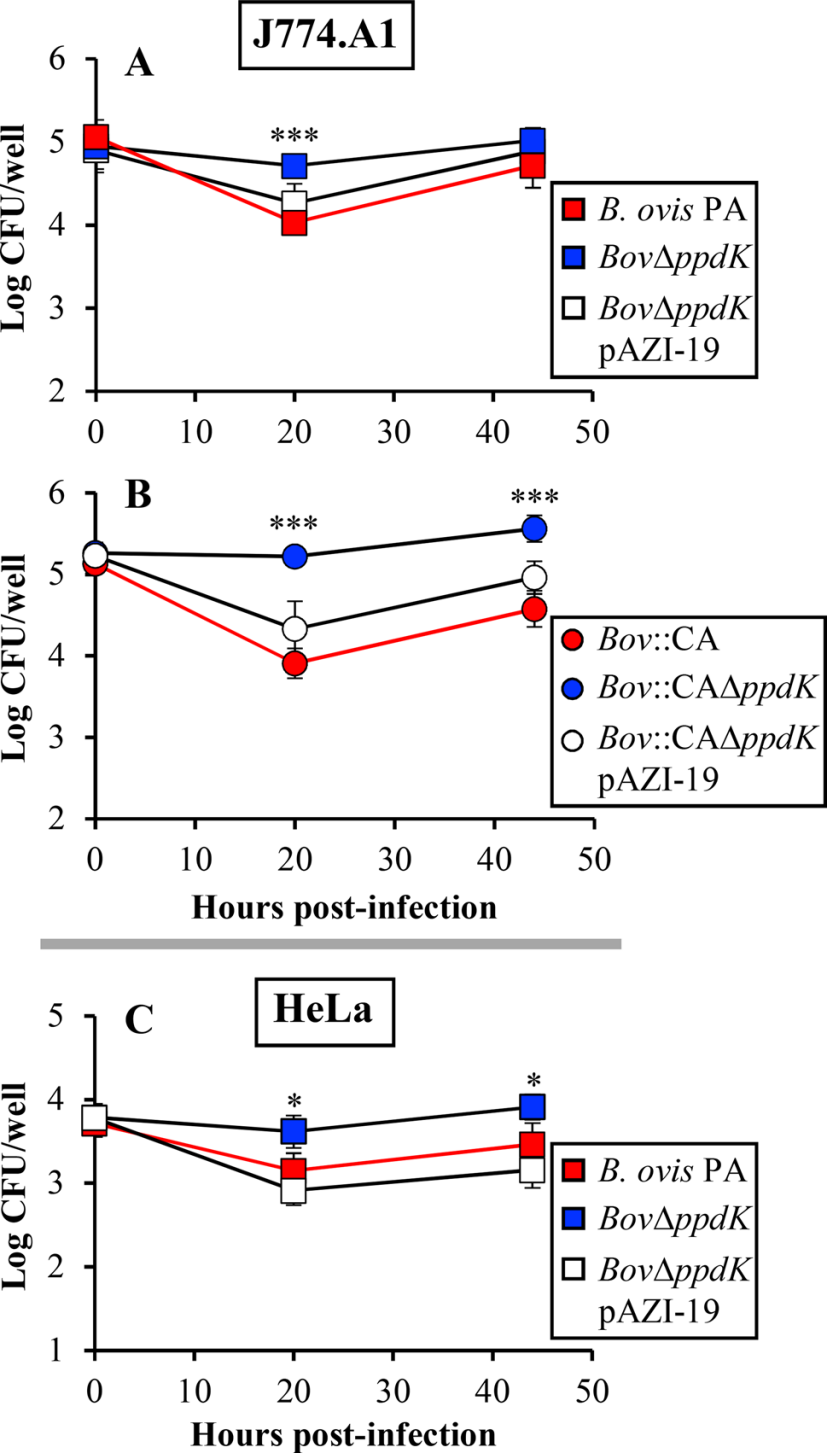
**Behaviour of the** ***B. ovis*** **strains in J774.A1 murine macrophages (A, B) and HeLa cells (C).** Cells were infected with CO_2_-dependent strains (**A**, **C**) or CO_2_-independent strains (**B**) and the intracellular CFU evaluated a t0, t20 and t44 as described in Materials and Methods. The results are expressed as mean ± SD of the log CFU/well and are representative of three independent experiments. Statistically significant differences (*P *< 0.01) with the corresponding parental strain are marked with asterisks (*P ≤0.01, **P ≤ 0.005; ***P ≤ 0.0005).

Since *B. ovis* is an intracellular pathogen, J774.A1 murine macrophages were used to determine the relevance of PpdK for intracellular survival and multiplication of *B. ovis* PA, *Bov*Δ*ppdK* (CO_2_-dependent) and *Bov*::CAΔ*ppdK* (CO_2_-independent) mutants, together with the respective parental strains and the mutant strains complemented with wild-type *ppdK*, were included in the study. No statistically significant differences of internalization in J744.A1 macrophages were observed among the CO_2_-dependent (Figure  2A) and -independent strains (Figure  2B). However, while the usual decrease [21, 40] of about 1 log unit in intracellular CFU numbers was observed after 20 h for the parental and complemented strains, both *ppdK* mutants maintained the intracellular values observed at the beginning of the experiment (Figure  2A, B). As expected, intracellular replication of parental and complemented strains was observed after 20 h (*P *< 0.0005), with intracellular CFU counts close to those at the beginning. Interestingly, although the *ppdK* mutants were not killed, their intracellular CFU numbers were similar at 20 and 44 h (Figure  2A, B), showing that they barely multiplied.

Considering the striking phenotype in macrophages, this part of the study was extended to evaluate the behavior of the CO_2_-dependent strains in a non-professional phagocyte (HeLa epithelial cells). In agreement with previous works with *B. ovis* PA [21, 40], the parental and complemented strains gave the expected pattern in HeLa cells (Figure  2C). As observed in J774.A1 macrophages, whereas intracellular counts of the *ppdK* mutant did not decrease at 20 h and were significantly higher (*P *< 0.01) than those of the parental strain, they did not increase significantly from 20 to 44 h (Figure  2C).

### Virulence in mice

**Figure 3:**
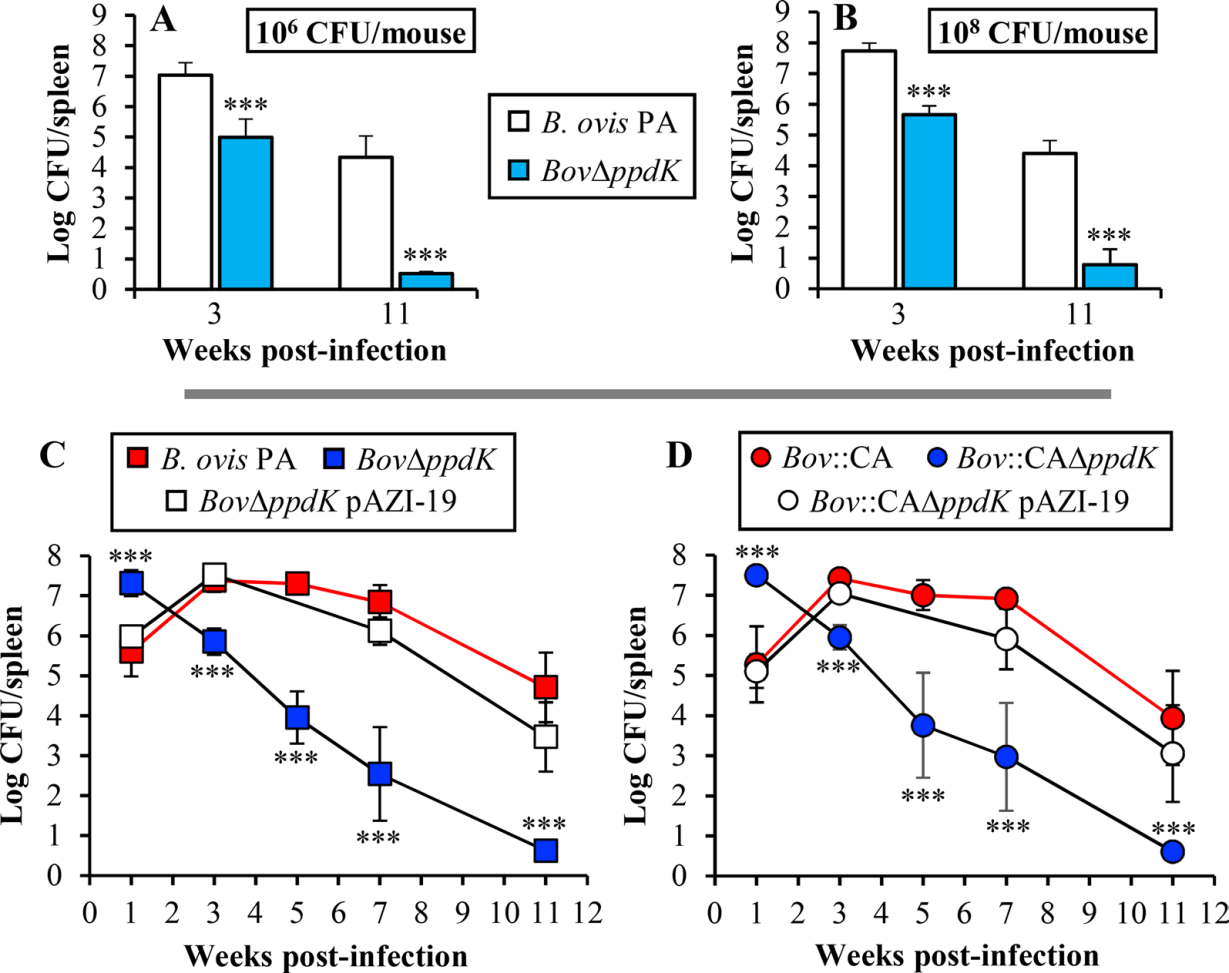
**Spleen colonization kinetics of strains derived from CO**_**2**_**-dependent (A–C) and CO**_**2**_**-independent (D)** ***B. ovis*** **PA**. In a first experiment (**A**, **B**), animals were inoculated with 10^6^ CFU (A) or 10^8^ CFU (B) of *B. ovis* PA or the *ppdK* mutant. The results are expressed as mean ± SD of the log CFU/spleen (n = 5) detected for each strain at weeks 3 and 11 p.i. In a second experiment (**C**, **D**), mice were inoculated with 10^8^ CFU of the CO_2_-dependent and CO_2_-independent strains and bacterial CFU were determined in spleen at weeks 1, 3, 5, 7 and 11 p.i. The results are expressed as mean ± SD of the log CFU/spleen (n = 5) detected for each strain. For each time point, statistically significant differences (*P* < 0.01), when compared to mice inoculated with the corresponding parental strain, are marked with asterisks (***P ≤ 0.0005).

In a first experiment, parental *B. ovis* PA and its *ppdK* mutant were inoculated in mice at 10^6^ or 10^8^ CFU/mouse to assess spleen colonization after 3 and 11 weeks. At p.i. week 3, mice inoculated with 10^6^ CFU of the *ppdK* mutant showed splenic counts c.a. 2 log units lower (*P *< 0.0005) than those of the parental strain (Figure  3A). At p.i. week 11 (Figure  3A), the attenuation of the *ppdK* mutant became more evident because, while the parental strain yielded splenic counts in the order of 4.5 log units, bacterial counts were beyond detection in the spleens of mice inoculated with the *ppdK* mutant (limit of detection of this method = 3.3 CFU/mL of the homogenized spleen [41]). Similar results were observed with the higher dose of inoculation, although in this case a few colonies of the *ppdK* mutant were detected in one mouse at p.i. week 11 (Figure  3B).

*Brucella* spleen replication profile in mice shows four phases: (i) onset or colonization phase (first 48 h); (ii) acute phase (from day 3 to weeks 2 to 4) where bacteria reach maximal numbers; (iii) chronic steady phase, where the bacterial numbers plateau; and (iv) chronic declining phase, when brucellae are progressively eliminated [41]. When intraperitoneally inoculated, and with small differences depending on the inoculation dose, *B. ovis* PA reaches the highest bacterial load in spleen after 3 to 4 weeks, the plateau phase continues until week 9 and then bacterial numbers decrease [17, 21, 42]. Using this model, a splenic colonization curve covering weeks 1 to 11 was obtained for all *B. ovis* strains inoculated at 10^8^ CFU/mouse. As previously reported, CO_2_-dependent and CO_2_-independent strains showed similar spleen counts throughout the experiment (Figure  3C, D) [28]. Surprisingly, both *ppdK* mutants yielded splenic CFU counts about 2 log units higher (*P *< 0.0005) than those observed for the respective parental strains at p.i. week 1 (Figure  3C, D). However, while the CFU of the parental strains increased at p.i. week 3 and maintained similar levels of infection until week 7, the splenic CFU of the *ppdK* mutants underwent a progressive decrease after week 1 and were not detected in any mouse at week 11 (Figure  3C, D). Complementation of the *ppdK* mutants with wild type *ppdK* restored phenotype of the parental strains (Figure  3C, D). Since these results show that both *ppdK* mutants failed to reach the chronic steady phase, they strongly suggest that PpdK activity plays a role during infection by wild-type *B. ovis* PA.

## Discussion

Although several works have identified genes encoding virulence factors in *B. ovis*, there is only indirect information on the metabolic abilities of this species and none on their connection with virulence. Thus, to gain insight into the metabolic properties of *B. ovis*, the enzymes related to PEP use were studied, a choice made here on the bases of their relevance in *B. abortus* 2308 W and *B. suis* 513 (reference strain of biovar 5) [26, 27]. Since no *Brucella* genome contains a gene encoding Pps (phosphoenolpyruvate synthase), the studied candidates were PckA (phosphoenolpyruvate carboxykinase) and PpdK (pyruvate phosphate dikinase). Previous studies have shown that while *B. suis* 513 relies on PpdK and PckA for PEP synthesis, *B. abortus* 2308 W uses only PpdK due to an inactivating frameshift in PckA. These observations have been interpreted to mean that some pathways or reactions remain functional in species or biovars that, like the rodent-associated *B. suis* 513, are close to the early diverging *Brucella* clades while becoming inactivated in species adapted to domestic livestock [26, 27]. The results obtained in this work are in line with this interpretation because they show that the picture in *B. ovis* PA is similar to that of *B. abortus* with an inactive PckA that is even shorter [26]. Why PpdK is conserved while PckA is not in some *Brucella* species might be related to the bidirectional nature of the enzymatic reaction catalyzed by PpdK in contrast to the role of PckA, which is involved only in the gluconeogenic direction. However, while both the genomic evidence and our experiments strongly suggest that *ppdK* encodes a functional enzyme catalyzing the pyruvate ⇌ phosphoenolpyruvate reaction, we have not been able to test this prediction because of the inability of *B. ovis* to grow on defined media containing only glutamate-lactate-glycerol as C sources, a strategy we have used previously to confirm this activity in *B. abortus* 2308 W and *B. suis* 513 [26, 27]. It is noteworthy that deletion of *ppdK* in these two smooth brucellae does not affect growth in rich medium (TSB) to any extent while the homologous deletion has this effect in *B. ovis* when growing on TSB-YE-HS. Indeed, it is not possible presently to identify the precise reason(s) for this difference because of the inability of *B. ovis* to grow on simple chemically defined media. This inability is remarkable because, in fact, Gerhardt’s medium supports growth of *B. abortus*, *B. melitensis* and *B. suis* biovar 1 better than other simple defined media, including those that contain glucose as C source, and this has been related both to the capability of many *Brucella* strains to carry out gluconeogenesis and to the ability of these bacteria to use glutamate very efficiently through the TCA. Also, the addition of serum and other nutritional complements to Gerhardt’s medium did not allow *B. ovis* growth, which means that potential nutrients in serum do not compensate for the complex amino acid and other nutrients present in peptones and yeast extract. This observation together with the impact of *ppdK* deletion in rich medium clearly shows that the metabolism of *B. ovis* differs from those of at least *B. abortus* 2308 W and *B. suis* 513, confirming that the internal diversity of the genus may be reflected in the metabolism [27]. Significantly, *B. ovis* is defective in the oxidative metabolism of arabinose, galactose, ribose, xylose, glucose, erythritol, fructose and mannose, but all *B. ovis* strains tested are able to oxidize adonitol, a carbohydrate not metabolized by *B. abortus*, *B. suis* and *B. melitensis* [43]. Tsolis et al. suggested that the metabolic defects of this species could be related to the inactivation by frameshifts, point mutations or gene degradation of several putative sugar transporters predicted to be functional in other *Brucella* species [38], as it is the case of the glucose/galactose transporter GluP, which is almost 100 bp shorter in *B. ovis* due to a point mutation [25].

The intracellular multiplication of the *ppdK* mutant in macrophages also showed differences with the behavior of the *B. abortus* 2308 W *ppdK* and *B. suis* 513 *pckA*-*ppdK* mutants. While these two mutants were less fit than the parental strains in the replicative niche and did not multiply to their level at 24 and 48 h post-infection [26 and unpublished results], *B. ovis* mutants showed intracellular counts at 20 h significantly higher than those of the parental and complemented strains. This behavior parallels the higher numbers of splenic counts detected at post-infection week 1 for both *ppdK* mutants. Moreover, the level of splenic infection progressively decreased thereafter, which could be related with the lack of significant multiplication of the mutants in phagocytes after 20 h. Resembling the phenotypes of the corresponding mutants in *B. abortus* 2308 W and *B. suis* 513, both *ppdK* mutants failed to reach the chronic steady phase typical of virulent brucellae, yielding significantly lower CFU counts after week 1. These results are compatible with the model proposed before for *B. abortus* 2308 W and *B. suis* 513 suggesting that in the replicative niche of these bacteria, glutamate, alanine, and other amino acids are important C sources, while a limited supply of 6 and 5 C would be used for biosynthesis of envelope polymers and for the Pentose Phosphate cycle-dependent biosynthetic reactions [26]. However, considering the above-discussed metabolic differences with the smooth brucellae, it is necessary to study other *B. ovis* mutants in key enzymes of the glycolytic and gluconeogenic pathways, or the anabolic routes bridging the TCA and the triose-phosphate pathway to support this hypothesis.

It is important to highlight the practical implications of this work. The kinetics of splenic infection observed with the of *B. ovis* PA *ppdK* mutants, with the high numbers of bacterial counts observed at week 1 and persistence until at least week 7, suggests that this mutant might be an interesting vaccine candidate against *B. ovis* infection. A similar profile of splenic infection in mice has been observed for another *B. ovis* attenuated mutant conferring homologous protection in the murine model, and with *B. melitensis* Rev1 [20], the classical vaccine used against *B. ovis* infection in sheep [10]. Moreover, *B. abortus* 2308 W and *B. suis* biovar 2 *ppdK* mutants showed good potential to immunize against virulent *B. abortus* and *B. suis*, respectively. In fact, both vaccine candidates reached the level of protection of the reference vaccines in the mouse model while showed reduced residual virulence (Zúñiga-Ripa et al., unpublished results and Aragón-Aranda et al., unpublished results). Therefore, the combination of *ppdK* deletion with the genetic modifications required for CO_2_-independent growth results in an interesting tool to study protection against sheep brucellosis caused by *B. ovis*.

## Supplementary information

**Additional file 1: Chemically defined media used in this work**.

**Additional file 2: (A) Amino acid sequence alignment of PckA in** ***A. tumefaciens*** **C58,** ***B. ovis*** **63/290,** ***B. abortus*** **2308 and** ***B. suis*** **513**. In bold and underlined, the specific domain (IGGTSYAGE-KKS; 190 to 202) required for the PckA activity, the phosphate-binding (G--G-GKT; 233 to 243) and adenine-binding (IIML—D; 345 to 351) consensus sites of ATP-dependent proteins and the metal ion binding (G----EG; 265 to 271) site. In red, amino acids (419-458) not conserved in *B. ovis* 63/290 due to the deletion of a cytosine at position 1523 of the *pckA* gene. **B** Amino acid sequence alignment of PpdK in *B. ovis* 63/290, *B. abortus* 2308 and *B. suis* 513. In red, the 3 amino acid changes found in *B. ovis* 63/290 in contrast to *B. abortus* 2308W and *B. suis* 513 (A177V, V312E, C857Y).

## Data Availability

The datasets used and/or analysed during the current study are available from the corresponding author on reasonable request.

## Abbreviations

AMP: adenosine monophosphate
ATP: adenosine triphosphate
HS: horse serum
KEGG: Kyoto Encyclopedia of Genes and Genomes
Km: kanamycin
LPS: lipopolysaccharide
Nal: nalidixic acid
NCBI: National Center for Biotechnology Information
PckA: phosphoenolpyruvate carboxykinase
PEP: phosphoenolpyruvate
P.i: post-infection
PpdK: pyruvate phosphate dikinase
Pps: phosphoenolpyruvate synthase
TCA: tricarboxylic Acid Cycle
TSA: tryptic soy agar
TSB: tryptic soy broth
YE: yeast extract
