# Supplementary material for: Disruption of pyruvate phosphate dikinase in Brucella ovis PA CO2-dependent and independent strains generates attenuation in the mouse model
Source: Vet Res. 2020 Aug 14;51:101. doi: 10.1186/s13567-020-00824-7 (PMC7427901; doi:10.1186/s13567-020-00824-7)
Supplement: Supplementary file 1 — Additional file 1: Chemically defined media used in this work. [file 13567_2020_824_MOESM1_ESM.pdf]

Components	Medium [1]			
	Gerhardt	Gerhardt-HS	Gerhardt-HS-meth	Gerhardt-HS-meth-vit
Organic macronutrients				
Glycerol	30.0 g	30.0 g	30.0 g	30.0 g
Lactic acid	5.0 g	5.0 g	5.0 g	5.0 g
Glutamic acid	1.5 g	1.5 g	1.5 g	1.5 g
Vitamins				
Thiamine	0.2 mg	0.2 mg	0.2 mg	0.2 mg
Nicotinic acid	0.2 mg	0.2 mg	0.2 mg	0.2 mg
Pantothenic acid	0.04 mg	0.04 mg	0.04 mg	0.04 mg
Biotin	0.0001 mg	0.0001 mg	0.0001 mg	0.0001 mg
Methionine (meth)	-	-	0.15 g	0.15 g
RPMI supplement R7256 (vit)	-	-	-	10 ml
Minerals				
K_2HPO_4	10.0 g	10.0 g	10.0 g	10.0 g
$Na_2S_2O_3 \cdot 5\ H_2O$	0.1 g	0.1 g	0.1 g	0.1 g
Mg^{2+} (as SO_4^{2-})	10.0 mg	10.0 mg	10.0 mg	10.0 mg
Mn^{2+} (as SO_4^{2-})	0.1 mg	0.1 mg	0.1 mg	0.1 mg
Fe^{2+} (as SO_4^{2-})	0.1 mg	0.1 mg	0.1 mg	0.1 mg
NaCl	7.5 g	7.5 g	7.5 g	7.5 g
Horse serum (HS)	-	50 ml	50 ml	50 ml

[1] Amounts per liter of broth
