# Supplementary material for: Disruption of pyruvate phosphate dikinase in Brucella ovis PA CO2-dependent and independent strains generates attenuation in the mouse model
Source: Vet Res. 2020 Aug 14;51:101. doi: 10.1186/s13567-020-00824-7 (PMC7427901; doi:10.1186/s13567-020-00824-7)
Supplement: Supplementary file 2 — Additional file 2: (A) Amino acid sequence alignment of PckA in A. tumefaciens C58, B. ovis 63/290, B. abortus 2308 and B. suis 513. In bold and underlined, the specific domain (IGGTSYAGE-KKS; 190 to 202) required for the PckA activity, the phosphate-binding (G--G-GKT; 233 to 243) and adenine-binding (IIML—D; 345 to 351) consensus sites of ATP-dependent proteins and the metal ion binding (G----EG; 265 to 271) site. In red, amino acids (419-458) not conserved in B. ovis 63/290 due to the deletion of a cytosine at position 1523 of the pckA gene. B Amino acid sequence alignment of PpdK in B. ovis 63/290, B. abortus 2308 and B. suis 513. In red, the 3 amino acid changes found in B. ovis 63/290 in contrast to B. abortus 2308W and B. suis 513 (A177V, V312E, C857Y). [file 13567_2020_824_MOESM2_ESM.pdf]

A

A. tumefaciens	MNELGVHNPANGVAELGLGEASRVFYNLNESELYEHAIRNGEAELTIDGALRAVTGQHTG	60
B. ovis	MKETGIHNKAASISTSGLKELSAVFYNLGAAWLYEETIRRGEAELSAQGALVARTGQHTG	60
B. abortus	MKETGIHNKAASISTSGLKELSAVFYNLGPARLYEETIRRGEAELSAQGALVARTGQHTG	60
B. suis	MKETGIHNKAASISTSGLKELSAVFYNLGPARLYEETIRRGEAELSAQGALVARTGQHTG	60
A. tumefaciens	RSPKDKFVVRDASTENTIWWDNNKPLSPENFELLRQDMLAHAAGKTLYVQDLIGGADEEN	120
B. ovis	RSPKDKFVVRDANTEDHVWWDNNKPMTPEAFELLYADFIEHAKGRELFVQDLIGGADADN	120
B. abortus	RSPKDKFVVRDANTEDHVWWDNNKPMTPEAFELLYADFIEHAKGRELFVQDLIGGADADN	120
B. suis	RSPKDKFVVRDANTEDHVWWDNNKPMTPEAFELLYADFIEHAKGRELFVQDLIGGADADN	120
A. tumefaciens	ALPTRVVTELAWHSLFIRNLLIRPKRETLSGFSQKLTIINLPSFKADPARHGVRSETVIA	180
B. ovis	KINARVITEYAWHSLFIRNLLIRPSQEALASYVPEMTIIDLPSFKADPERYGVRTETVIA	180
B. abortus	KINARVITEYAWHSLFIRNLLIRPSQEALASYVPEMTIIDLPSFKADPERYGVRTETVIA	180
B. suis	KINARVITEYAWHSLFIRNLLIRPSQEALASYVPEMTIIDLPSFKADPERYGVRTETVIA	180
A. tumefaciens	CDLTNGLVL**IGGTSYAGE**N**KKS**VFTVLNYLLPAKGVMPMHCSANVGPEGDSAVFF**G**LS**G**T	240
B. ovis	VDLTRKIVL**IGGTSYAGE**M**KKS**VFTALNYILPAKGVMPMHCSANEGPNGDTAVFF**G**LS**G**T	240
B. abortus	VDLTRKIVL**IGGTSYAGE**M**KKS**VFTALNYILPAKGVMPMHCSANEGPNGDTAVFF**G**LS**G**T	240
B. suis	VDLIRKIVL**IGGTSYAGE**M**KKS**VFTALNYILPAKGVMPMHCSANEGPNGDTAVFF**G**LS**G**T	240
A. tumefaciens	**GKT**TLSADPARTLIGDDEHGWGEH**G**IFNF**EG**GCYAKAIKLSSEAEPEIYAATNRFGTVLE	300
B. ovis	**GKT**TLSADPTRTLIGDDEHGWGEH**G**VFNF**EG**GCYAKTIRLSAEAEPEIYATTQRFGTVLE	300
B. abortus	**GKT**TLSADPTRTLIGDDEHGWGEH**G**VFNF**EG**GCYAKTIRLSAEAEPEIYATTQRFGTVLE	300
B. suis	**GKT**TLSADPTRTLIGDDEHGWGEH**G**VFNF**EG**GCYAKTIRLSAEAEPEIYATTQRFGTVLE	300
A. tumefaciens	NVVLDESRVPDFNDNSLTENTRSAYPLHFIPNASETGIAGHPKT**IIML**TA**D**AFGVLPPIA	360
B. ovis	NVVLDENRQPDFDDGSLTENTRCAYPLDFIPNASKSGKGGQPKN**IIML**TA**D**AFGVMPPIA	360
B. abortus	NVVLDENRQPDFDDGSLTENTRCAYPLGFIPNASKSGKGGQPKN**IIML**TA**D**AFGVMPPIA	360
B. suis	NVVLDENRQPDFDDGSLTENTRCAYPLDFIPNASKSGKGGQPKN**IIML**TA**D**AFGVMPPIA	360
A. tumefaciens	RLTPEQAMYHFLSGYTAKVAGTEKGVTEPEATFSTCFGAPFMPRHPAEYGNLLRELIGKH	420
B. ovis	KLTPAQAMYHFLSGYTAKVAGTEKGVTEPEATFSTCFGAPFMPRHPSEYGNLLRKLIA**ST**	420
B. abortus	KLTPAQAMYHFLSGYTAKVAGTEKGVTEPEATFSTCFGAPFMPRHPSEYGNLLRKLIAEH	420
B. suis	KLTPAQAMYHFLSGYTAKVAGTEKGVTEPEATFSTCFGAPFMPRHPSEYGNLLRKLIAEH	420
A. tumefaciens	G-VDCWLVNTGWTGG---AYGIGKRMPIKATRALLTAALTGDLKNAQFRTDANFGFAVPL	476
B. ovis	**RLIAGWSIPAGRAALTALASGCRSRQPAR**------------------**FWQRPSTAR**----	458
B. abortus	KV-DCWLVNTGWTGG---AYGVGKRMPIKATRALLAAALDGSLNNAEFRIDPNFGFAVPV	476
B. suis	KV-DCWLVNTGWTGG---AYGVGKRMPIKATRALLAAALDGSLNNAEFRIDPNFGFAVPV	476
A. tumefaciens	SLDGVDGAILDPRSTWADGAAYDAQAKKLVSMFVSNFTKFEDHVDSKVRDAAPGLLLAAE	536
B. ovis	--	458
B. abortus	EVPGVESSILDPRST---	491
B. suis	EVPGVESSILDPRSTWADKVAYDAQAKKLVDMFVSNFEKFESHVDHEVKDAAPAIRMAAE	536

B

B. ovis	MAKWVYTFGDGKAEGAASDRNLLGGKGANLAEMSSLGLPVPPGFTITTEVCTYYYNNDRV	60
B. abortus	MAKWVYTFGDGKAEGAASDRNLLGGKGANLAEMSSLGLPVPPGFTITTEVCTYYYNNDRV	60
B. suis	MAKWVYTFGDGKAEGAASDRNLLGGKGANLAEMSSLGLPVPPGFTITTEVCTYYYNNDRV	60
B. ovis	YPSELDAQVQAALAHIATLTGRNFGDAEKPLLVSVRSGARASMPGMMDTVLNLGLNDETV	120
B. abortus	YPSELDAQVQAALAHIATLTGRNFGDAEKPLLVSVRSGARASMPGMMDTVLNLGLNDETV	120
B. suis	YPSELDAQVQAALAHIATLTGRNFGDAEKPLLVSVRSGARASMPGMMDTVLNLGLNDETV	120
B. ovis	QAIARESGDERFAYDSYRRFIQMYSDVVLGVDHGFFEEILEDTKADLGVEVDTALS**V**DDW	180
B. abortus	QAIARESGDERFAYDSYRRFIQMYSDVVLGVDHGFFEEILEDTKADLGVEVDTALS**A**DDW	180
B. suis	QAIARESGDERFAYDSYRRFIQMYSDVVLGVDHGFFEEILEDTKADLGVEVDTALS**A**DDW	180
B. ovis	KNVIGLYKAKVEEELGQPFPQDPREQLWGAIGAVFSSWMNARAITYRRLHNIPAAWGTAV	240
B. abortus	KNVIGLYKAKVEEELGQPFPQDPREQLWGAIGAVFSSWMNARAITYRRLHNIPAAWGTAV	240
B. suis	KNVIGLYKAKVEEELGQPFPQDPREQLWGAIGAVFSSWMNARAITYRRLHNIPAAWGTAV	240
B. ovis	NVQAMVFGNMGETSATGVAFTRNPSTGENKLYGEFLVNAQGEDVVAGIRTPQNITEEARI	300
B. abortus	NVQAMVFGNMGETSATGVAFTRNPSTGENKLYGEFLVNAQGEDVVAGIRTPQNITEEARI	300
B. suis	NVQAMVFGNMGETSATGVAFTRNPSTGENKLYGEFLVNAQGEDVVAGIRTPQNITEEARI	300
B. ovis	AAGSDKPSLEK**E**MPEAFAEFLKVANRLEQHYRDMQDLEFTIERGKLWMLQTRSGKRTARA	360
B. abortus	AAGSDKPSLEK**V**MPEAFAEFLKVANRLEQHYRDMQDLEFTIERGKLWMLQTRSGKRTARA	360
B. suis	AAGSDKPSLEK**V**MPEAFAEFLKVANRLEQHYRDMQDLEFTIERGKLWMLQTRSGKRTARA	360
B. ovis	ALKMAVEMAAEGLISEEEAVLRIDPAALDQLLHPTIDPRAERQVVGMGLPASPGAATGEI	420
B. abortus	ALKMAVEMAAEGLISEEEAVLRIDPAALDQLLHPTIDPRAERQVVGMGLPASPGAATGEI	420
B. suis	ALKMAVEMAAEGLISEEEAVLRIDPAALDQLLHPTIDPRAERQVVGMGLPASPGAATGEI	420
B. ovis	VFSSEEAEQAKAEGRNVILVRIETSPEDIHGMHAAEGILTTRGGMTSHAAVVARGMGKPC	480
B. abortus	VFSSEEAEQAKAEGRNVILVRIETSPEDIHGMHAAEGILTTRGGMTSHAAVVARGMGKPC	480
B. suis	VFSSEEAEQAKAEGRNVILVRIETSPEDIHGMHAAEGILTTRGGMTSHAAVVARGMGKPC	480
B. ovis	VSGAGSLRVDYRNGTMLAAGQTFRKGDVITIDGASGQVLKGSVAMLQPELSGDFGKLMEW	540
B. abortus	VSGAGSLRVDYRNGTMLAAGQTFRKGDVITIDGASGQVLKGSVAMLQPELSGDFGKLMEW	540
B. suis	VSGAGSLRVDYRNGTMLAAGQTFRKGDVITIDGASGQVLKGSVAMLQPELSGDFGKLMEW	540
B. ovis	ADRARRMKVRANAETPADARTARSFGAEGIGLCRTEHMFFDGSRIVAMREMILSDTEEGR	600
B. abortus	ADRARRMKVRANAETPADARTARSFGAEGIGLCRTEHMFFDGSRIVAMREMILSDTEEGR	600
B. suis	ADRARRMKVRANAETPADARTARSFGAEGIGLCRTEHMFFDGSRIVAMREMILSDTEEGR	600
B. ovis	RLALAKLLPMQRSDFAELFEIMKGLPVTIRLLDPPLHEFLPHTDEEVDEVARSMGVDAAK	660
B. abortus	RLALGKLLPMQRSDFAELFEIMKGLPVTIRLLDPPLHEFLPHTDEEVDEVARSMGVDAAK	660
B. suis	RLALAKLLPMQRSDFAELFEIMKGLPVTIRLLDPPLHEFLPHTDEEVDEVARSMGVDAAK	660
B. ovis	LRDRADALHEFNPMLGHRGCRLAVSYPEIAEMQARAIFEAAVEAGKKTGEPVVPEVMVPL	720
B. abortus	LRDRADALHEFNPMLGHRGCRLAVSYPEIAEMQARAIFEAAVEAGKKTGEPVVPEVMVPL	720
B. suis	LRDRADALHEFNPMLGHRGCRLAVSYPEIAEMQARAIFEAAVEAGKKTGEPVVPEVMVPL	720
B. ovis	VGLKAELDFVKARIDAVAKEVMSEAGIKIDYMVGTMIELPRAALRAAEIAESAEFFSFGT	780
B. abortus	VGLKAELDFVKARIDAVAKEVMSEAGIKIDYMVGTMIELPRAALRAAEIAESAEFFSFGT	780
B. suis	VGLKAELDFVKARIDAVAKEVMSEAGIKIDYMVGTMIELPRAALRAAEIAESAEFFSFGT	780
B. ovis	NDLTQTTFGISRDDAAGFLTTYQNRGVIEQDPFVSLDVDGVGELVQIAAERGRKTREKIK	840
B. abortus	NDLTQTTFGISRDDAAGFLTTYQNRGVIEQDPFVSLDVDGVGELVQIAAERGRKTREKIK	840
B. suis	NDLTQTTFGISRDDAAGFLTTYQNRGVIEQDPFVSLDVDGVGELVQIAAERGRKTREKIK	840
B. ovis	LGICGEHGGDPASIAF**Y**EKTGLDYVSCSPFRVPIARLAAAQAAVRKV	887
B. abortus	LGICGEHGGDPASIAF**C**EKTGLDYVSCSPFRVPIARLAAAQAAVRKV	887
B. suis	LGICGEHGGDPASIAF**C**EKTGLDYVSCSPFRVPIARLAAAQAAVRKV	887
